# Longitudinal serum bicarbonate and mortality risk in older patients with advanced chronic kidney disease: analyses from the EQUAL cohort

**DOI:** 10.1093/ckj/sfae254

**Published:** 2024-08-22

**Authors:** Gianmarco Lombardi, Nicholas C Chesnaye, Fergus J Caskey, Friedo W Dekker, Marie Evans, Olof Heimburger, Maria Pippias, Claudia Torino, Maciej Szymczak, Christiane Drechsler, Christoph Wanner, Giovanni Gambaro, Vianda S Stel, Kitty J Jager, Pietro Manuel Ferraro, the EQUAL study investigators, Andreas Schneider, Anke Torp, Beate Iwig, Boris Perras, Christian Marx, Christiane Drechsler, Christof Blaser, Christoph Wanner, Claudia Emde, Detlef Krieter, Dunja Fuchs, Ellen Irmler, Eva Platen, Hans Schmidt-Gürtler, Hendrik Schlee, Holger Naujoks, Ines Schlee, Sabine Cäsar, Joachim Beige, Jochen Röthele, Justyna Mazur, Kai Hahn, Katja Blouin, Katrin Neumeier, Kirsten Anding-Rost, Lothar Schramm, Monika Hopf, Nadja Wuttke, Nikolaus Frischmuth, Pawlos Ichtiaris, Petra Kirste, Petra Schulz, Sabine Aign, Sandra Biribauer, Sherin Manan, Silke Röser, Stefan Heidenreich, Stephanie Palm, Susanne Schwedler, Sylke Delrieux, Sylvia Renker, Sylvia Schättel, Theresa Stephan, Thomas Schmiedeke, Thomas Weinreich, Til Leimbach, Torsten Stövesand, Udo Bahner, Wolfgang Seeger, Adamasco Cupisti, Adelia Sagliocca, Alberto Ferraro, Alessandra Mele, Alessandro Naticchia, Alex Còsaro, Andrea Ranghino, Andrea Stucchi, Angelo Pignataro, Antonella De Blasio, Antonello Pani, Aris Tsalouichos, Bellasi Antonio, Biagio Raffaele Di Iorio, Butti Alessandra, Cataldo Abaterusso, Chiara Somma, Claudia D'alessandro, Claudia Torino, Claudia Zullo, Claudio Pozzi, Daniela Bergamo, Daniele Ciurlino, Daria Motta, Domenico Russo, Enrico Favaro, Federica Vigotti, Ferruccio Ansali, Ferruccio Conte, Francesca Cianciotta, Francesca Giacchino, Francesco Cappellaio, Francesco Pizzarelli, Gaetano Greco, Gaetana Porto, Giada Bigatti, Giancarlo Marinangeli, Gianfranca Cabiddu, Giordano Fumagalli, Giorgia Caloro, Giorgina Piccoli, Giovanbattista Capasso, Giovanni Gambaro, Giuliana Tognarelli, Giuseppe Bonforte, Giuseppe Conte, Giuseppe Toscano, Goffredo Del Rosso, Irene Capizzi, Ivano Baragetti, Lamberto Oldrizzi, Loreto Gesualdo, Luigi Biancone, Manuela Magnano, Marco Ricardi, Maria Di Bari, Maria Laudato, Maria Luisa Sirico, Martina Ferraresi, Michele Provenzano, Moreno Malaguti, Nicola Palmieri, Paola Murrone, Pietro Cirillo, Pietro Dattolo, Pina Acampora, Rita Nigro, Roberto Boero, Roberto Scarpioni, Rosa Sicoli, Rosella Malandra, Silvana Savoldi, Silvio Bertoli, Silvio Borrelli, Stefania Maxia, Stefano Maffei, Stefano Mangano, Teresa Cicchetti, Tiziana Rappa, Valentina Palazzo, Walter De Simone, Anita Schrander, Bastiaan van Dam, Carl Siegert, Carlo Gaillard, Charles Beerenhout, Cornelis Verburgh, Cynthia Janmaat, Ellen Hoogeveen, Ewout Hoorn, Friedo Dekker, Johannes Boots, Henk Boom, Jan-Willem Eijgenraam, Jeroen Kooman, Joris Rotmans, Kitty Jager, Liffert Vogt, Maarten Raasveld, Marc Vervloet, Marjolijn van Buren, Merel van Diepen, Nicholas Chesnaye, Paul Leurs, Pauline Voskamp, Peter Blankestijn, Sadie van Esch, Siska Boorsma, Stefan Berger, Constantijn Konings, Zeynep Aydin, Aleksandra Musiała, Anna Szymczak, Ewelina Olczyk, Hanna Augustyniak-Bartosik, Ilona Miśkowiec-Wiśniewska, Jacek Manitius, Joanna Pondel, Kamila Jędrzejak, Katarzyna Nowańska, Łukasz Nowak, Maciej Szymczak, Magdalena Durlik, Szyszkowska Dorota, Teresa Nieszporek, Zbigniew Heleniak, Andreas Jonsson, Anna-Lena Blom, Björn Rogland, Carin Wallquist, Denes Vargas, Emöke Dimény, Fredrik Sundelin, Fredrik Uhlin, Gunilla Welander, Isabel Bascaran Hernandez, Knut-Christian Gröntoft, Maria Stendahl, Maria Svensson, Marie Evans, Olof Heimburger, Pavlos Kashioulis, Stefan Melander, Tora Almquist, Ulrika Jensen, Alistair Woodman, Anna McKeever, Asad Ullah, Barbara McLaren, Camille Harron, Carla Barrett, Charlotte O'Toole, Christina Summersgill, Colin Geddes, Deborah Glowski, Deborah McGlynn, Dympna Sands, Fergus Caskey, Geena Roy, Gillian Hirst, Hayley King, Helen McNally, Houda Masri-Senghor, Hugh Murtagh, Hugh Rayner, Jane Turner, Joanne Wilcox, Jocelyn Berdeprado, Jonathan Wong, Joyce Banda, Kirsteen Jones, Lesley Haydock, Lily Wilkinson, Margaret Carmody, Maria Weetman, Martin Joinson, Mary Dutton, Michael Matthews, Neal Morgan, Nina Bleakley, Paul Cockwell, Paul Roderick, Phil Mason, Philip Kalra, Rincy Sajith, Sally Chapman, Santee Navjee, Sarah Crosbie, Sharon Brown, Sheila Tickle, Suresh Mathavakkannan, Ying Kuan

**Affiliations:** Division of Nephrology, Department of Medicine, University of Verona and Azienda Ospedaliera Universitaria Integrata, Verona, Italy; ERA Registry, Amsterdam UMC location University of Amsterdam, Medical Informatics, Amsterdam, The Netherlands; Amsterdam Public Health Research Institute, Quality of Care, Amsterdam, The Netherlands; Population Health Sciences, Bristol Medical School, University of Bristol, Bristol, UK; Department of Clinical Epidemiology, Leiden University Medical Center, Leiden, The Netherlands; Renal Unit, Department of Clinical Intervention and Technology, Karolinska Institutet and Karolinska University Hospital, Stockholm, Sweden; Renal Unit, Department of Clinical Intervention and Technology, Karolinska Institutet and Karolinska University Hospital, Stockholm, Sweden; Population Health Sciences, Bristol Medical School, University of Bristol, Bristol, UK; North Bristol NHS Trust, Renal Unit, Bristol, UK; Institute of Clinical Physiology-National Research Council, Clinical Epidemiology and Pathophysiology of Renal Diseases and Hypertension, Reggio Calabria, Italy; Department of Nephrology and Transplantation Medicine, Wroclaw Medical University, Wroclaw, Poland; Department of Clinical Research and Epidemiology, University Hospital Würzburg, Würzburg, Germany; Department of Clinical Research and Epidemiology, University Hospital Würzburg, Würzburg, Germany; Division of Nephrology, Department of Medicine, University of Verona and Azienda Ospedaliera Universitaria Integrata, Verona, Italy; ERA Registry, Amsterdam UMC location University of Amsterdam, Medical Informatics, Amsterdam, The Netherlands; Amsterdam Public Health Research Institute, Quality of Care, Amsterdam, The Netherlands; ERA Registry, Amsterdam UMC location University of Amsterdam, Medical Informatics, Amsterdam, The Netherlands; Amsterdam Public Health Research Institute, Quality of Care, Amsterdam, The Netherlands; Division of Nephrology, Department of Medicine, University of Verona and Azienda Ospedaliera Universitaria Integrata, Verona, Italy

**Keywords:** bicarbonate, chronic kidney disease, elderly, mortality

## Abstract

**Background:**

We aimed to explore the relationship between serum bicarbonate (SBC) and mortality in advanced chronic kidney disease (CKD) during three distinct treatment periods: during the pre-kidney replacement therapy (KRT) period, during the transition phase surrounding the start of KRT (transition-CKD) and during KRT.

**Methods:**

Using the European QUALity Study on treatment in advanced CKD (EQUAL) cohort, which includes patients aged ≥65 years and estimated glomerular filtration rate (eGFR) ≤20 mL/min/1.73 m^2^ from six European countries, we explored the association between longitudinal SBC and all-cause mortality in three separate CKD populations: pre-KRT, transition-CKD and in the KRT populations, using multivariable time-dependent Cox regression models. We evaluated effect modification by pre-specified variables on the relationship between SBC and mortality.

**Results:**

We included 1485 patients with a median follow-up of 2.9 (interquartile range 2.7) years, during which 529 (35.6%) patients died. A U-shaped relationship between SBC levels and all-cause mortality was observed in the pre-KRT population (*P* = .03). Low cumulative exposure, defined as the area under the SBC trajectory before KRT initiation, was associated with increased mortality risk after transitioning to KRT (*P* = .01). Similarly, in the KRT population, low SBC levels showed a trend towards increased mortality risk (*P* = .13). We observed effect modification by subjective global assessment category (*P*-value for interaction = .02) and KRT (*P*-value for interaction = .02).

**Conclusions:**

A U-shaped relationship describes the association between SBC and mortality in the advanced CKD pre-KRT population, whereas in the KRT population a trend towards an increased mortality risk was observed for low SBC levels.

KEY LEARNING POINTS **What was known:**

- Serum bicarbonate (SBC) levels are linked to mortality in chronic kidney disease (CKD) patients.
- Studies have predominantly focused on pre-kidney replacement therapy (KRT) or post-KRT phases, neglecting to explore the continuum from pre-KRT to post-KRT initiation.
- Optimal management of SBC levels in advanced CKD patients remains uncertain.

**This study adds:**

- Reveals a U-shaped relationship between SBC and mortality in pre-KRT CKD patients and highlights the role of KRT initiation on the relationship between SBC and mortality.
- Highlights the varying associations of SBC with mortality across different CKD stages, showing that this relationship is modified by KRT initiation, thus providing a clearer understanding of this complex relationship.
- Emphasizes the need for personalized management of acid–base balance disorders based on a comprehensive evaluation of the clinical status of the patient.

**Potential impact:**

- Offers insights for personalized management of acid–base balance in CKD patients, potentially influencing clinical practice.
- May inform guidelines on maintaining SBC levels, optimizing care for advanced CKD patients.
- Could prompt further research on interventions targeting metabolic acidosis in CKD patients, shaping future treatment strategies.

## INTRODUCTION

Serum bicarbonate (SBC) is an important buffer of the human body which helps maintain the acid–base balance [1]. It is produced by the kidneys, and its levels are regulated by a complex interplay of factors, including acid–base status, diet and medication use [2, 3]. Chronic metabolic acidosis is among the most common chronic kidney disease (CKD)-related complications and a well-recognized modifiable risk factor for mortality [2]. Decreases in renal ammonium excretion and a positive acid balance are the main mechanisms responsible for metabolic acidosis usually observed in the CKD population [2].

SBC disorders have been consistently linked to mortality in CKD patients [4–11]. Low SBC levels have been extensively associated with increased mortality in CKD patients, possibly due to the detrimental effects of acidosis on various organs and systems [7]. Likewise, several studies have identified high levels of SBC as a risk factor for mortality in the CKD population [5, 9]. The precise mechanisms underlying this effect have not been established, although an impact on the presence or severity of underlying comorbid conditions is a possibility [5], as well as a direct harmful effect of metabolic alkalosis *per se* on myocardium, skeletal muscle and the central nervous system [12, 13].

To date, most studies have focused on the association between SBC levels and patient survival during the pre-kidney replacement therapy (KRT) period [5, 6] or the period after KRT initiation [14, 15], considered separately. None has explored this association across the continuum of CKD (e.g. from pre-KRT to post-KRT start), which is relevant since metabolic acidosis is expected to be managed through dialysis in the KRT population, and thus its clinical outcomes might change after KRT initiation. In order to provide new insights on this topic, we aim to investigate the association between SBC and mortality risk in older individuals with advanced CKD across three distinct clinical phases: the pre-KRT period, the KRT phase and the transition phase surrounding the start of KRT.

## MATERIALS AND METHODS

### Study design and population

**Figure 1:**
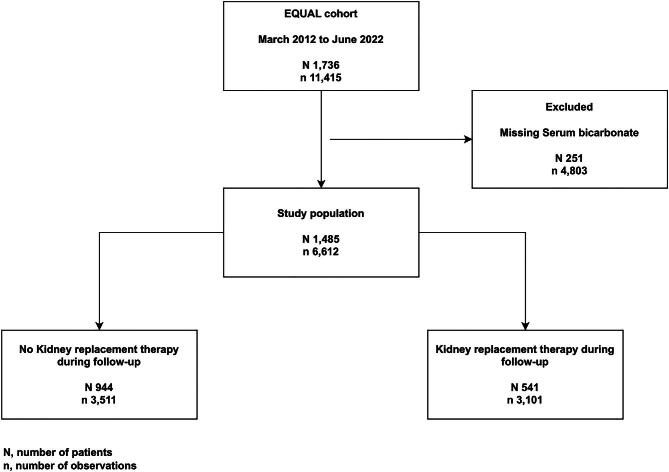
Flowchart of the study population.

The European QUALity Study on treatment in advanced CKD (EQUAL) is an ongoing observational multicenter cohort study including CKD patients of 65 years of age and older, with an incident estimated glomerular filtration rate (eGFR) <20 mL/min/1.73 m² not on KRT, receiving routine medical care in Germany, Italy, the Netherlands, Poland, Sweden and the UK. Participants were excluded if the drop in eGFR resulted from an acute event or if they had previously received KRT or a kidney transplant. Study visits and data collection were scheduled at 3- to 6-month intervals, and participants were followed until kidney transplantation, death, refusal for further participation, or loss to follow-up. Approval was obtained from the medical ethical committees in each country. Informed consent was obtained from all participants. A full description of the study has been published elsewhere [16]. For this particular study, starting from a source population of 1736 patients between March 2012 and June 2022, we included all patients with at least one available measurement of SBC (Fig. 1).

### Data collection

Clinical data were collected on demographics, primary kidney disease, laboratory data, medication, nutritional status, lifestyle and comorbid conditions. eGFR was calculated from serum creatinine level standardized to isotope dilution mass spectrometry using the 2009 Chronic Kidney Disease Epidemiology Collaboration (CKD-EPI) equation [17]. Primary kidney disease was classified using the European Renal Association codes [18].

### Exposure and outcome of interest

**Figure 2:**
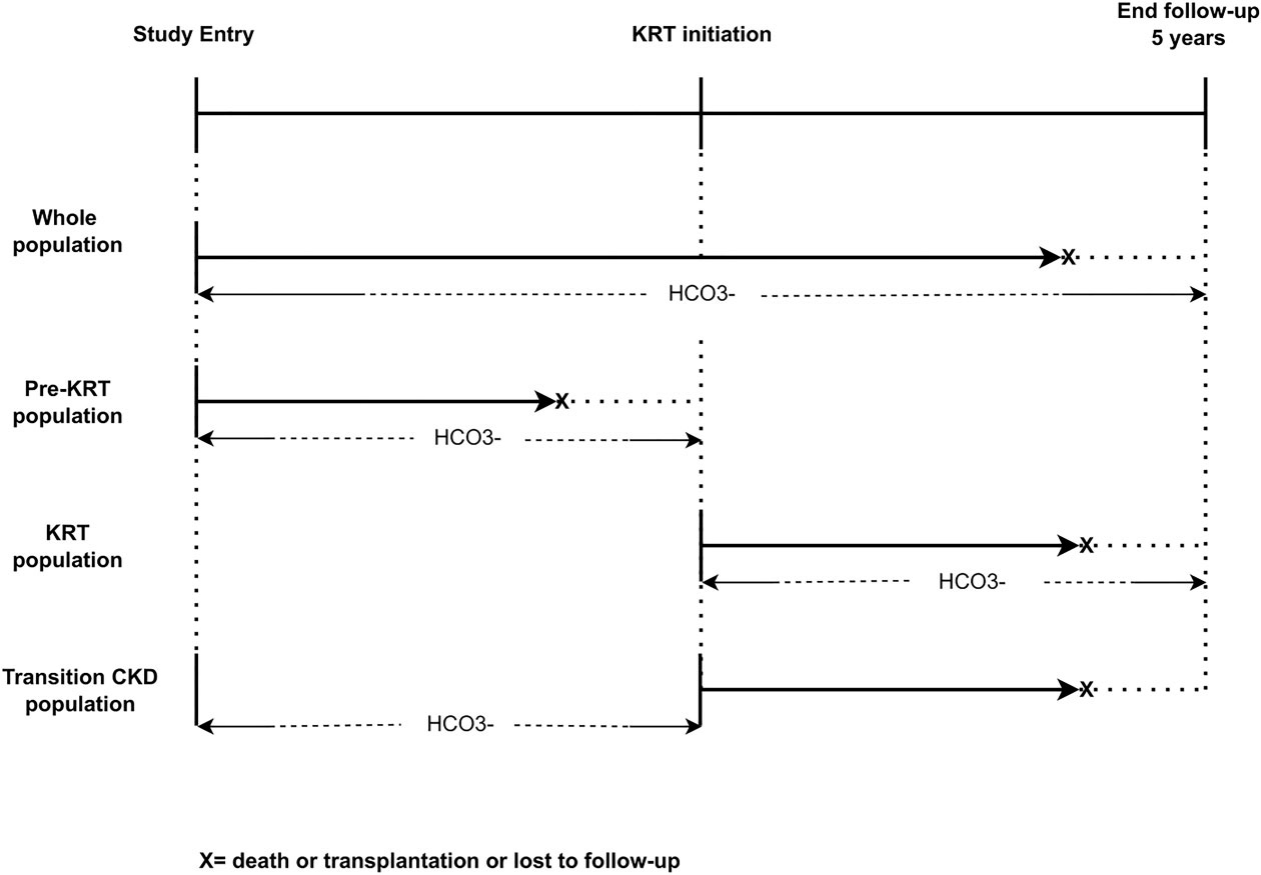
Study design.

All-cause mortality was the primary outcome of interest. Time-updated SBC during follow-up was the exposure of interest. Effect modification by KRT on the association between longitudinal SBC and mortality risk was explored in three different populations. (i) In the entire EQUAL population (‘whole population’), where we also tested for an interaction effect between longitudinal SBC and KRT initiation on mortality risk. (ii) In the entire EQUAL population, we assessed the association between longitudinal SBC and mortality risk in pre-KRT patients by censoring at KRT initiation (‘pre-KRT population’). (iii) In those that started KRT, we assessed the association between longitudinal SBC and mortality risk, using KRT start as baseline (‘KRT population’). (iv) In those that started KRT, transitioning from the pre-KRT phase to the KRT phase (‘transition-CKD population’). In this population we also assessed the association between SBC and mortality risk, using KRT start as baseline, but now evaluating the cumulative effect of the SBC exposure history up to the point of KRT initiation. To achieve this, the cumulative time-averaged, the cumulative exposure of SBC and the slope of the SBC trajectory during the pre-KRT phase were calculated (Fig. 2). The SBC slope was calculated using linear regression in patients with two or more SBC measurements and was categorized by quartiles. As visits were sometimes planned at irregular time intervals, before calculating cumulative time-averaged SBC we generated a balanced dataset using a linear mixed model to extrapolate SBC measurements at every 6 months during the time period prior to KRT initiation for each patient. Cumulative time-averaged SBC exposure was calculated as the ratio between the cumulative sum of all SBC measurements before KRT initiation and the number of visits for each patient. A linear mixed model was used to impute missing SBC values at 6-month time points. To determine the cumulative exposure of SBC during the pre-KRT phase we calculated the area under the curve of the SBC levels over time, from the study entry to the KRT initiation, using the trapezoidal rule by a sum of the integrals defined by the SBC values at each time point and for each time interval of the SBC measurement [19].

### Statistical analysis

Continuous variables were reported using the mean and standard deviation (SD) for normally distributed variables, or the median and interquartile range (IQR) if skewed. Categorical variables were reported using frequencies and percentages. Categorical variables were compared using the chi-square test. Continuous variables were compared using Student’s *t-*test or Mann–Whitney test as appropriate. Normality of distribution was assessed by inspecting Q-Q plots and histograms.

Time-dependent Cox regression models were used to estimate the association between longitudinal SBC (both continuous and categorized at baseline by quartiles of baseline SBC) and mortality risk. SBC, body mass index (BMI), albumin, subjective global assessment (SGA) and serum potassium (K^+^) were treated as time-dependent variables. Natural cubic splines were used to describe the continuous relationship between SBC and all-cause mortality in all populations. We conducted a Kaplan–Meier survival analysis on the outcome of interest comparing quartiles of baseline SBC. The log-rank test was used to test for differences in survival. Survival time was defined as the time from the first study visit or KRT initiation (according to study population) to death, transplantation, loss to follow-up or end of follow-up at 5 years.

We used five levels of adjustment in our models: Model 1, an unadjusted Cox regression model; Model 2, adjusted for sex and age; Model 3, Model 2 plus BMI, albumin and SGA; Model 4, Model 3 plus baseline eGFR and K^+^; and Model 5, Model 4 plus primary kidney diseases and comorbidities (diabetes mellitus, cerebrovascular disease, peripheral vascular disease, coronary artery disease, myocardial infarction, heart failure, left ventricular hypertrophy, malignancy, psychiatric disease, connective tissue disorders and respiratory diseases).

Subgroup analyses were performed to evaluate effect modification on the relationship between SBC and mortality risk. The study population was stratified into the following subgroups: age (≤70 or >70 years old), sex, BMI (≤25 or >25 kg/m^2^), albumin (≤35 or >35 g/L), SGA (≤6 or >6), comorbidities (cardiovascular diseases, diabetes, malignancies, respiratory diseases, connective tissue disorders).

Because of the well-documented influence of K^+^ on SBC concentrations [1] and their effect on mortality, the interaction between them was tested separately in the whole population.

As a sensitivity analysis, the relationship between longitudinal SBC and mortality risk across subgroups was also assessed in a time-dependent Cox regression model, adjusted for all covariates previously reported (Model 5) plus medications used (treated as time-varying variables). The following medications were included in the models: diuretic, sodium bicarbonate, renin–angiotensin systems inhibitors and beta-blockers.

Furthermore, multivariable-adjusted time-dependent mixed Cox regression models were used to evaluate and compare different slopes in the log hazard of death for specific values of continuous SBC levels. SBC was modelled as a natural spline with 3 degrees of freedom. Given the repeated measurements for each patient, we employed a mixed-effects model with patient ID as a random intercept to account for intra-patient correlation. We set SBC values by dividing the SBC distribution into three equal parts (percentiles P25, P50 and P75). At each value, we estimated the slope, indicating the change in the log hazard of death associated with a 1 unit increase of SBC at that particular value. Adjustments for multiple comparisons between slopes were made using the Tukey method. The same analyses were performed for each subgroup of interest.

At baseline, missing values in the covariates were handled by replacing the missing values with either the mode (categorical variable) or median (continuous variable) of the respective variable. Longitudinal missing data were then imputed using the last observation carried forward method. A *P*-value <.05 was considered statistically significant. All analyses were performed in R version 4.2.1 (R Core Team, Vienna, Austria).

## RESULTS

### Descriptive characteristics of the study population

Out of 1736 EQUAL patients, 1485 matched our inclusion criteria (Fig. 1). Of those, 541 (36.4%) initiated KRT during follow-up. Baseline characteristics of the study population stratified by quartile of SBC are reported in Table 1. Median age of the study population was 76.0 (IQR 10.7) years with 33.4% being women. Patients in the higher quartile (SBC >25.5 mmol/L) were older, with a higher prevalence in women. Notably, a higher prevalence of most cardiovascular diseases was observed in the highest quartile. A higher prevalence in sodium bicarbonate therapy was observed in the first SBC quartile, and a higher prevalence in diuretic therapy was reported in the last quartile (Table 1).

**Table 1:** Baseline characteristics. of the study population.

		Quartile (Q) of SBC				
	Overall: *N* = 1485, *n* = 6612	1st (<20.5 mmol/L): *N* = 371, *n* = 1449	2nd (20.5–22.9 mmol/L): *N* = 350, *n* = 1602	3rd (23.0–25.5 mmol/L): *N* = 391, *n* = 1904	4th (>25.5 mmol/L): *N* = 373, *n* = 1657	*P*-value
Demographic data						
Age, years, median (IQR)	76.0 (10.7)	74.6 (11.4)	75.9 (10.8)	76.3 (10.8)	77.0 (10.3)	.003
Sex, female, *N* (%)	495 (33.4)	96 (25.9)	113 (32.4)	132 (33.8)	154 (41.6)	<.001
Country, *N* (%)						<.001
Germany	121 (8.1)	27 (7.3)	28 (8.0)	29 (7.4)	37 (9.9)	
Italy	378 (25.5)	82 (22.1)	76 (21.7)	90 (23.0)	130 (34.9)	
Netherlands	249 (16.8)	45 (12.1)	54 (15.4)	78 (19.9)	72 (19.3)	
Poland	86 (5.8)	44 (11.9)	32 (9.1)	7 (1.8)	3 (0.8)	
Sweden	174 (11.7)	38 (10.2)	53 (15.1)	55 (14.1)	28 (7.5)	
UK	477 (32.1)	135 (36.4)	107 (30.6)	132 (33.8)	103 (27.6)	
Level of education, *N* (%)						.002
None/low	383 (32.7)	90 (31.1)	83 (29.7)	99 (31.8)	111 (37.8)	
Intermediate	570 (48.6)	126 (43.6)	144 (51.6)	160 (51.4)	140 (47.6)	
High	169 (14.4)	64 (22.1)	39 (14.0)	39 (12.5)	27 (9.2)	
Other	51 (4.3)	9 (3.1)	13 (4.7)	13 (4.2)	16 (5.4)	
Examination data						
SBP, mmHg, mean (SD)	141.9 (22.0)	143.6 (21.7)	143.3 (21.4)	142.0 (21.9)	138.8 (22.9)	.013
DBP, mmHg, mean (SD)	73.3 (11.3)	74.7 (11.1)	74.2 (10.8)	73.1 (11.2)	71.3 (12.0)	<.001
BMI, kg/m^2^, median (IQR)	27.9 (6.6)	27.3 (7.0)	27.8 (6.5)	28.2 (6.0)	28.3 (7.3)	.082
Urea, mmol/L, median (IQR)	19.5 (9.0)	21.4 (9.9)	19.2 (8.2)	18.7 (8.2)	19.3 (9.4)	<.001
Bicarbonate, mmol/L, mean (SD)	23.1 (4.0)	18.2 (2.0)	21.6 (0.6)	24.1 (0.8)	28.2 (2.4)	<.001
K^+^, mmol/L, mean (SD)	4.7 (0.6)	4.9 (0.7)	4.7 (0.6)	4.6 (0.6)	4.5 (0.6)	<.001
Albumin, g/L, median (IQR)	38.4 (6.6)	38.0 (7.0)	39.0 (5.7)	39.0 (6.0)	38.0 (7.0)	<.001
eGFR, mL/min/1.73 m^2^, median (IQR)	16.7 (6.7)	15.1 (6.5)	16.1 (6.6)	17.6 (6.4)	17.4 (7.0)	<.001
SGA, median (IQR)	6.0 (1.0)	5.9 (1.2)	6.1 (1.0)	6.1 (1.0)	5.9 (1.0)	.012
Primary renal disease, *N* (%)						.093
Glomerular disease	124 (8.4)	41 (11.2)	28 (8.1)	27 (6.9)	28 (7.6)	
Tubulo-interstitial disease	129 (8.8)	38 (10.4)	37 (10.7)	29 (7.4)	25 (6.8)	
Diabetic kidney disease	309 (21.0)	68 (18.5)	65 (18.7)	94 (24.1)	82 (22.3)	
Hypertension	531 (36.1)	119 (32.4)	120 (34.6)	147 (37.7)	145 (39.5)	
Other/unknown	378 (25.7)	101 (27.5)	97 (28.0)	93 (23.8)	87 (23.7)	
Comorbidities, *N* (%)						
Hypertension	1250 (88.8)	317 (89.8)	297 (89.5)	332 (89.0)	304 (87.1)	.676
Diabetes	621 (42.8)	143 (39.5)	139 (40.9)	168 (43.3)	171 (47.4)	.155
Cerebrovascular disease	224 (15.6)	53 (14.6)	44 (13.1)	60 (15.5)	67 (18.9)	.184
Peripheral vascular disease	233 (16.3)	53 (14.7)	51 (15.1)	60 (15.8)	69 (19.5)	.286
Coronary artery disease	383 (27.1)	83 (23.4)	90 (26.7)	107 (28.3)	103 (29.9)	.249
Myocardial infarction	258 (17.8)	57 (15.7)	61 (18.0)	73 (18.8)	67 (18.7)	.670
Heart failure	239 (17.0)	47 (13.4)	45 (13.6)	61 (16.2)	86 (24.8)	<.001
Left ventricular hypertrophy	324 (25.2)	77 (24.1)	62 (20.9)	92 (25.9)	93 (29.4)	.105
Malignancies	304 (21.2)	70 (19.3)	78 (23.6)	91 (23.7)	65 (18.2)	.150
Psychiatric diseases	94 (6.5)	25 (6.9)	15 (4.4)	25 (6.4)	29 (8.1)	.265
Connective tissue disorders	41 (2.8)	8 (2.2)	10 (2.9)	13 (3.4)	10 (2.8)	.821
Respiratory diseases	225 (15.5)	44 (12.1)	48 (14.2)	53 (13.7)	80 (22.3)	.001
Medications, *N* (%)						
Vitamin D active	28 (1.9)	5 (1.4)	5 (1.4)	8 (2.1)	10 (2.7)	.504
Phosphate binders calcium based	15 (1.0)	5 (1.4)	3 (0.9)	3 (0.8)	4 (1.1)	.862
Phosphate binders calcium free	27 (1.8)	8 (2.2)	7 (2.0)	6 (1.5)	6 (1.6)	.903
Calcimimetic	0 (0.0)	0 (0.0)	0 (0.0)	0 (0.0)	0 (0.0)	
RAASi	100 (6.8)	21 (5.7)	26 (7.5)	22 (5.6)	31 (8.4)	.333
Beta-blocker	112 (7.6)	23 (6.2)	21 (6.1)	24 (6.2)	44 (12.0)	.004
Statin	84 (5.7)	16 (4.3)	19 (5.5)	21 (5.4)	28 (7.6)	.272
Diuretic	140 (9.5)	23 (6.2)	30 (8.7)	39 (10.0)	48 (13.1)	.015
Sodium bicarbonate	52 (3.5)	17 (4.6)	13 (3.8)	8 (2.1)	14 (3.8)	.272

Missing data at baseline: 0.3% age, 0.3% sex, 21.0% level of education, 2.3% SBP, 2.3% DBP, 9.2% BMI, 3.0% urea, 0.3% K^+^, 8.4% albumin, 0.1% eGFR, 14.5% SGA, 0.9% primary renal disease, 2.3% diabetes, 3.1% cerebrovascular disease, 3.8% peripheral vascular disease, 4.7% coronary artery disease, 2.4% myocardial infarction, 5.4% heart failure, 13.4% left ventricular hypertrophy, 3.4% malignancies, 2.4% psychiatric diseases, 2.4% connective tissue disorders, 2.4% lung diseases, 0.9% medications.

eGFR is by CKD-EPI equation.

RAASi, renin–angiotensin–aldosterone system inhibitors; SBP, systolic blood pressure; DBP, diastolic blood pressure; SGA, subjective global assessment; K^+^, serum potassium; *N*, number of patients; *n*, number of observations; SD, standard deviation.

### SBC and all-cause mortality in the whole population

**Figure 3:**
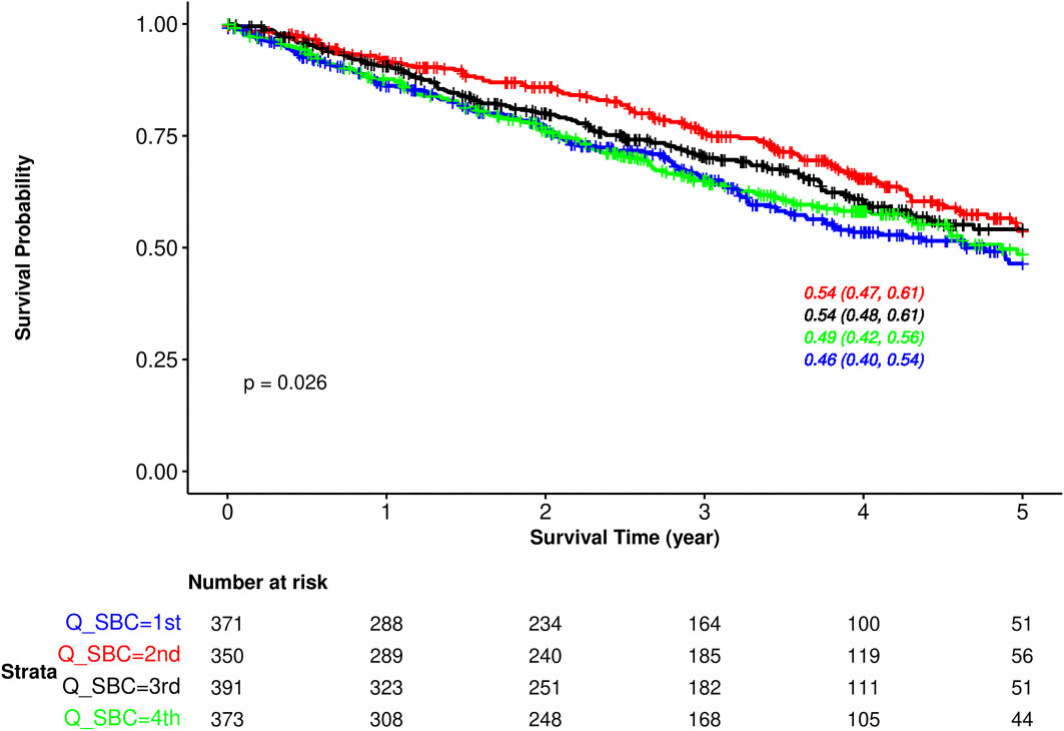
Kaplan–Meier survival curves for all-cause mortality based on quartiles of baseline SBC in the whole population. Results are displayed up to 5 years. The overall 5-year survival was 51% (95% CI 47, 54). Q_SBC, quartile of serum bicarbonate.

**Figure 4:**
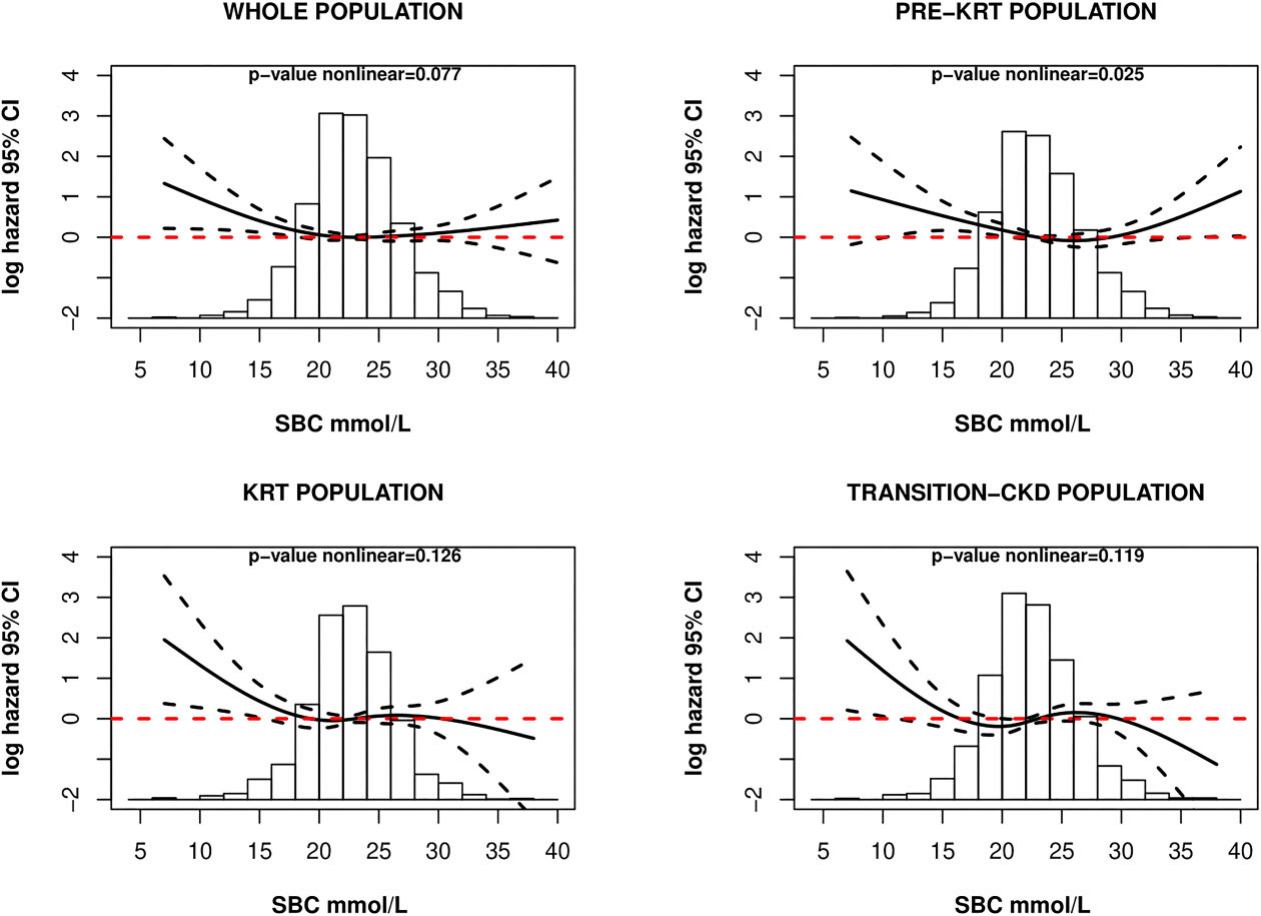
Continuous relationship between SBC and all-cause mortality in all study populations. Multivariable adjusted model, adjusted to age, sex, BMI, SGA, albumin, K^+^, eGFR, primary renal disease and comorbidities. Whole population, *P*-value nonlinear = .077; pre-KRT population, *P*-value nonlinear = .025; KRT population, *P*-value nonlinear = .126; transition-CKD population, *P*-value nonlinear = .119.

During a median follow-up of 2.9 (IQR 2.7) years, 529 (35.6%) patients died; cardiovascular mortality was the main cause of death (Supplementary data, Table S1). Kaplan–Meier analysis showed significant differences in all-cause mortality by quartile of baseline SBC (log-rank *P* = .03; Fig. 3). The unadjusted survival at 5 years was 46% [95% confidence interval (CI) 40, 54] for the first quartile of baseline SBC, 54% (95% CI 47, 61) for the second quartile, 54% (95% CI 48, 61) for the third quartile and 49% (95% CI 42, 56) for the fourth quartile (Fig. 3). An increased hazard of death, although not statistically significant, was observed in the lowest and highest quartiles of SBC (Table 2). Analysis of SBC as a continuous (longitudinal) variable revealed a J-shaped relationship, although statistical significance was not reached (adjusted *P*-value = .08) (Fig. 4). Similar results were observed in sensitivity analysis adjusting for medications use (Supplementary data, Fig. S1). The estimated slopes at SBC levels of 15, 23 and 31 mmol/L are presented in Supplementary data, Fig. S2, suggesting an increased risk of mortality with decreasing SBC levels below 15 mmol/L (–0.34, 95% CI –0.58, –0.10, *P* = .005).

**Table 2:** Relationship between longitudinal bicarbonate levels and all-cause mortality in the whole cohort (whole population).

Q SBC^a^	Events, *N* (%)	Person time, years	Events for 1000 person-years	Unadjusted, HR (95% CI)	Sex, age, HR (95% CI)^b^	BMI, albumin, SGA, HR (95% CI)^b^	eGFR, K^+^, HR (95% CI)^b^	Comorbidities, primary renal disease, HR (95% CI)^b^
1st	146 (39.4)	989.7	148	1.23 (0.97, 1.57); *P* = .093	1.27 (1, 1.62); *P* = .055	1.2 (0.94, 1.54); *P* = .141	1.22 (0.95, 1.57); *P* = .124	1.21 (0.93, 1.58); *P* = .158
2nd	110 (31.4)	1042.3	106	1 (0.78, 1.28); *P* = .970	0.99 (0.77, 1.27); *P* = .911	1.03 (0.8, 1.33); *P* = .806	1.04 (0.81, 1.33); *P* = .779	1.04 (0.81, 1.35); *P* = .751
3rd	129 (33.0)	1074.8	120	1.00 (Reference)	1.00 (Reference)	1.00 (Reference)	1.00 (Reference)	1.00 (Reference)
4th	144 (38.6)	1030.0	140	1.17 (0.93, 1.49); *P* = .178	1.16 (0.91, 1.47); *P* = .225	1.1 (0.86, 1.4); *P* = .466	1.09 (0.85, 1.39); *P* = .507	1.08 (0.85, 1.39); *P* = .517

Time-dependent Cox regression model; overall study population *N* = 1485.

^a^Quartiles of serum bicarbonate; SBC, BMI, albumin, SGA, K^+^ and eGFR treated as time-varying covariates.

^b^HR and 95% CI adjusted sequentially for confounders (sex, age, BMI, SGA, eGFR, K^+^, comorbidities, primary renal disease).

### SBC and all-cause mortality in the pre-KRT population

Out of 1457 patients included in the pre-KRT population, 345 (23.7%) patients died during a median follow-up of 2.0 (IQR 2.5) years; the most common cause of cardiovascular mortality was death due to heart failure (Supplementary data, Table S1).

A significantly increased hazard of death was observed in the lowest quartile of SBC in the fully adjusted model [hazard ratio (HR) 1.51, 95% CI 1.07, 2.11, *P* = .018; Table 3]. Analysis of SBC as a continuous (longitudinal) variable revealed a U-shaped relationship, with increasing mortality risk for both lower and higher SBC values (adjusted *P*-value = .025; Fig. 4). Similar results were observed in sensitivity analysis adjusting for medications use (Supplementary data, Fig. S1). A significant risk difference was found between the slope in the log hazard of death at 15 mmol/L and 31 mmol/L of SBC (15–31 mmol/L: –0.47, 95% CI –0.85, –0.08, *P* = 0.012), while the differences between the other SBC values were not statistically significant (Supplementary data, Fig. S2).

**Table 3:** Relationship between longitudinal bicarbonate levels and all-cause mortality censoring at KRT initiation (pre-KRT population).

Q SBC^a^	Events, *N* (%),	Person time, years	Events for 1000 person-years	Unadjusted, HR (95% CI)	Sex, age, HR (95% CI)^b^	BMI, albumin, SGA, HR (95% CI)^b^	eGFR, K^+^, HR (95% CI)^b^	Comorbidities, primary renal disease, HR (95% CI)^b^
1st	82 (22.8)	683.4	120	1.52 (1.11, 2.06); *P* = .008	1.58 (1.16, 2.16); *P* = .004	1.46 (1.06, 2); *P* = .019	1.46 (1.06, 2.02); *P* = .022	1.51 (1.07, 2.11); *P* = .018
2nd	70 (20.3)	764.7	92	1.16 (0.85, 1.59); *P* = .346	1.16 (0.84, 1.59); *P* = .368	1.17 (0.85, 1.61); *P* = .328	1.17 (0.85, 1.61); *P* = .336	1.18 (0.85, 1.63); *P* = .334
3rd	88 (22.9)	853.5	103	1.00 (Reference)	1.00 (Reference)	1.00 (Reference)	1.00 (Reference)	1.00 (Reference)
4th	105 (28.5)	823.6	127	1.35 (1, 1.82); *P* = .051	1.31 (0.97, 1.77); *P* = .081	1.22 (0.89, 1.66); *P* = .214	1.22 (0.89, 1.67); *P* = .222	1.19 (0.87, 1.63); *P* = .284

Time-dependent Cox regression model; overall study population *N* = 1457.

^a^Quartiles of serum bicarbonate; SBC, BMI, albumin, SGA, K^+^ and eGFR treated as time-varying covariates.

^b^HR and 95% CI adjusted sequentially for confounders (sex, age, BMI, SGA, eGFR, K^+^, comorbidities, primary renal disease).

### SBC and all-cause mortality in the transition-CKD population

Out of 502 patients included in the transition-CKD population, 177 (35.2%) patients died during a median follow-up of 3.8 (IQR 2.6) years. SBC levels before and after KRT initiation were quite similar (Supplementary data, Fig. S3).

In the transition-CKD population, no differences were observed among SBC quartiles and the hazard of death (Table 4). When analysing SBC as a continuous exposure, only patients with lower SBC levels tended to have a higher mortality risk, although statistical significance was not reached (adjusted *P*-value = .119; Fig. 4). Similar results were observed in sensitivity analysis adjusting for medications use (Supplementary data, Fig. S1).

**Table 4:** Relationship between longitudinal bicarbonate levels and all-cause mortality in the transition-CKD population.

Q SBC^a^	Events, *N* (%)	Person time, years	Events for 1000 person-years	Unadjusted, HR (95% CI)	Sex, age, HR (95% CI)^b^	BMI, albumin, SGA, HR (95% CI)^b^	eGFR, K^+^, HR (95% CI)^b^	Comorbidities, primary renal disease, HR (95% CI)^b^
1st	60 (39.0)	474.0	127	0.82 (0.54, 1.23); *P* = .336	0.84 (0.56, 1.26); *P* = .391	0.86 (0.57, 1.29); *P* = .458	0.87 (0.58, 1.32); *P* = .514	0.88 (0.57, 1.36); *P* = .572
2nd	39 (28.9)	489.4	80	0.76 (0.5, 1.15); *P* = .188	0.74 (0.49, 1.13); *P* = .161	0.85 (0.56, 1.29); *P* = .451	0.86 (0.57, 1.31); *P* = .485	0.9 (0.59, 1.39); *P* = .640
3rd	40 (36.0)	386.1	104	1.00 (Reference)	1.00 (Reference)	1.00 (Reference)	1.00 (Reference)	1.00 (Reference)
4th	38 (37.3)	355.7	107	1.05 (0.71, 1.55); *P* = .819	1.05 (0.71, 1.57); *P* = .791	0.92 (0.61, 1.39); *P* = .687	0.89 (0.59, 1.36); *P* = .601	0.92 (0.61, 1.38); *P* = .685

Time-dependent Cox regression model; overall study population *N* = 502.

^a^Quartiles of serum bicarbonate; SBC, BMI, albumin, SGA, K^+^ and eGFR treated as time-varying covariates.

^b^HR and 95% CI adjusted sequentially for confounders (sex, age, BMI, SGA, eGFR, K^+^, comorbidities, primary renal disease).

No differences were observed among quartiles of pre-KRT cumulative time-averaged of SBC and mortality risk (Supplementary data, Table S2). Similarly, no differences were observed among quartiles of individual SBC slopes and mortality risk (Supplementary data, Table S3 and Fig. S4).

A significant association was observed between low cumulative SBC exposure before KRT initiation and mortality risk. Lower quartiles of the area under the trajectory of pre-KRT SBC were significantly associated with increase hazard of death (Supplementary data, Table S4), which remained consistent when the relationship was explored analysing the area under the trajectory of pre-KRT SBC as a continuous exposure (Supplementary data, Fig. S4).

### SBC and all-cause mortality in the KRT population

Out of 441 patients included in the KRT population, 176 (39.9%) patients died during a median follow-up of 1.9 (IQR 2.4) years. Compared with the previous populations, a higher proportion of cardiovascular mortality due to cardiac arrest was observed after KRT initiation, as well as an increase in death due to infectious disease (Supplementary data, Table S1).

When comparing quartiles of SBC and mortality, no increased risk was observed (Table 5). These findings remained consistent when the relationship was explored analysing SBC as a continuous exposure (adjusted *P*-value = .126; Fig. 4). However, a trend towards an increased mortality risk was observed for low SBC levels. Similar results were observed in sensitivity analysis adjusting for medications use (Supplementary data, Fig. S1). Significant differences were found between the slopes in the log hazard of death at 15 mmol/L and 23 mmol/L of SBC (15–23 mmol/L: –0.57, 95% CI –1.09, –0.05, *P* = .027), while the differences between the other SBC values were not statistically significant (Supplementary data, Fig. S2).

**Table 5:** Relationship between longitudinal bicarbonate levels and all-cause mortality after KRT initiation (KRT population).

Q SBC^a^	Events, *N* (%)	Person time, years	Events for 1000 person-years	Unadjusted, HR (95% CI)	Sex, age, HR (95% CI)^b^	BMI, albumin, SGA, HR (95% CI)^b^	eGFR, K^+^, HR (95% CI)^b^	Comorbidities, primary renal disease, HR (95% CI)^b^
1st	50 (40.3)	258.6	193	1.21 (0.81, 1.81); *P* = .343	1.18 (0.79, 1.77); *P* = .407	1.24 (0.83, 1.85); *P* = .287	1.28 (0.85, 1.91); *P* = .233	1.2 (0.77, 1.85); *P* = .42
2nd	36 (38.3)	217.9	165	0.88 (0.57, 1.36); *P* = .574	0.87 (0.56, 1.34); *P* = .521	0.93 (0.6, 1.43); *P* = .736	0.95 (0.61, 1.47); *P* = .811	0.88 (0.55, 1.38); *P* = .567
3rd	41 (37.3)	252.0	163	1.00 (Reference)	1.00 (Reference)	1.00 (Reference)	1.00 (Reference)	1.00 (Reference)
4th	49 (43.4)	257.2	191	1 (0.67, 1.49); *P* = .999	0.99 (0.66, 1.48); *P* = .961	0.91 (0.59, 1.39); *P* = .654	0.89 (0.58, 1.37); *P* = .602	0.94 (0.62, 1.45); *P* = .788

Time-dependent Cox regression model; overall study population *N* = 441.

^a^Quartiles of serum bicarbonate; SBC, BMI, albumin, SGA and K^+^ treated as time-varying covariates.

^b^HR and 95% CI adjusted sequentially for confounders (sex, age, BMI, SGA, eGFR, K^+^, comorbidities, primary renal disease).

### SBC and all-cause mortality in subgroups

We observed significant effect modification on the relationship between continuous SBC and all-cause mortality by SGA (*P*-value for interaction = .02; Supplementary data, Fig. S5A) and KRT (*P*-value for interaction = .02; Supplementary data, Fig. S5B). Patients with SGA score ≤6 displayed a stronger association between lower SBC and mortality risk. Consistent with our previous findings, patients who initiated KRT showed a significant association between lower SBC and the log-hazard of death. On the other hand, in the subgroup that did not initiate KRT, a U-shaped association between SBC and death was observed. No significant interaction was observed between K^+^ and SBC (*P*-value for interaction = .41).

## DISCUSSION

In the current article, we performed a comprehensive analysis on the association between longitudinal SBC and all-cause mortality in an international multicenter cohort of older advanced CKD patients, across the continuum of advanced CKD stages, from the pre-KRT phase to the post-KRT. We observed a U-shaped relationship between SBC and mortality in the pre-KRT population, where both low and high SBC values were associated with an increased risk of mortality. In the KRT population, a trend towards an increased mortality risk was observed for low SBC levels. Low cumulative SBC exposure before transitioning to KRT tended to correlate with higher mortality risk post-KRT initiation; however, our findings were inconsistent across various statistical approaches. Differences in nutritional factors, comorbidities and cause-specific mortality might play a role in explaining these findings.

The U-shaped relationship between SBC and mortality in non-dialysis-dependent CKD aligns with several previous studies. Research conducted by Navaneethan *et al*. [5], as well as the study by Kovesdy *et al*. [9], consistently reported that both low and high longitudinal measurements of SBC levels were associated with an increased risk of mortality among non-dialysis-dependent CKD patients, even after adjusting for confounding factors such as age, sex and comorbidities.

This complex relationship between SBC and mortality among CKD patients is not yet fully understood. CKD patients often experience metabolic acidosis, characterized by low SBC levels, which can lead to adverse effects such as bone disease, muscle wasting and cardiovascular complications, all of which increase the risk of mortality [2, 20, 21]. Additionally, metabolic acidosis can trigger insulin resistance and dyslipidemia, further increasing mortality risk in CKD patients [22].

On the other hand, elevated SBC levels have also been linked to an increased risk of mortality in non-dialysis-dependent CKD patients. These findings may be influenced by comorbidities such as heart failure or respiratory diseases [5], commonly associated with metabolic alkalosis. Analysis from the Chronic Renal Insufficiency Cohort (CRIC) study point out the association of high SBC levels with cardiovascular diseases, reporting a 14% increased risk of heart failure for every 1 mmol/L increase in carbon dioxide above 24 mmol/L [23]. The high prevalence that we observed of cardiovascular comorbidities and respiratory diseases in patients in the higher quartiles of SBC levels are not surprising and consistent with prior studies.

Unlike the U-shaped relationship observed in the pre-KRT population, upon KRT initiation, we observed a trend towards an increased mortality risk for low SBC levels. A more pronounced inflammatory state and increased oxidative stress, commonly observed after the initiation of KRT, may underlie the connection between low SBC levels and mortality. From this point of view, it is not surprising to observe a higher prevalence of mortality due to infectious diseases in this group. However, it is worth noting that KRT significantly improves SBC correction, making it easier to identify patients with more severe metabolic acidosis, which is associated with a higher mortality risk compared with mild acidosis forms. Although there have been mixed data regarding the relationship between bicarbonate and mortality in the KRT population (e.g. DOPPS data suggest that the U-shaped curve best represents the relationship between SBC and mortality [11]), the majority of evidence from large observational cohorts supports and confirms our findings [8, 14].

In addition to the stage of CKD (before or after the initiation of KRT), nutritional factors also appear to play an important role in the relationship between SBC and mortality. We observed effect modification of patient nutritional status, as measured by SGA score, on the relationship between SBC and death, finding a stronger association between low SBC levels and death in the lower SGA score subgroup. This suggests that malnourished and catabolic patients may be more susceptible to the negative effects of low SBC levels. This finding is consistent with previous research showing that low SBC levels are commonly seen in malnourished and catabolic patients [21, 24]. Additionally, low SBC levels can also negatively impact the nutrition status of CKD patients, directly inducing the inflammatory system [24, 25].

Focusing on the transition-CKD population we observed an association between low SBC levels and mortality risk after KRT initiation. However, our findings were inconsistent across several statistical approaches. This finding has only been addressed to date in the work of Tantisattamo *et al*., conducted within the context of the Transition Care in CKD cohort, where the average 6-month time-average levels of SBC correlated with mortality in a U-shaped relationship [10]. A notable deviation from the prior study is observed in the varied duration of SBC exposure considered for evaluating SBC levels, with our study showing a median duration of 4.2 years. The exposure to low SBC levels may have influenced the progression of kidney disease, malnutrition and muscle wasting, thereby potentially contributing to the increased mortality observed following the initiation of KRT.

Several limitations of our study must be addressed. It is worth noting that due to the observational nature of our study, we cannot establish a causal relationship between SBC levels and mortality. Residual confounding may be present in our study, as we were unable to control for all potential confounding factors that may be associated with both SBC levels and mortality risk. Our approach of dividing the population based on dialysis status, as well as our quartiles analyses, may have decreased statistical power, limiting our ability to detect significant differences between the groups we were studying. The lack of data on the exact timing of the blood sampling for pre-KRT SBC may have influenced our results. Pre-KRT SBC levels are known to be significantly higher on midweek, after the short-interval KRT. Furthermore, it is important to note that the relationship between SBC and mortality in the transition CKD population may be subject to selection bias since only patients that remained alive up until the point of KRT initiation were included.

Our study also has several strengths—it is the first to examine the association between longitudinal SBC and mortality in older advanced CKD patients, crossing KRT initiation, providing a more comprehensive and clear understanding of this complex relationship. This has also allowed us to further highlight the role of KRT initiation on the relationship between SBC and mortality. In addition, by analysing the whole study population, before and after KRT initiation, in a European setting, we give more generalizability to our results.

The 2021 KDIGO Clinical Practice Guideline for Evaluation and Management of CKD recommend that SBC levels are maintained at or above 22–32 mmol/L and suggest sodium bicarbonate use to maintain SBC levels ≥22 mmol/L [26]. However, to date, the evidence regarding the use of sodium bicarbonate in patients with CKD is still limited and conflicting [27–30].

Our study has provided important insights into the relationship between SBC and mortality in advanced CKD. Our findings suggest that the relationship between SBC and mortality varies across different stages of CKD and that this relationship is modified by KRT initiation.

From this point of view, it would be appropriate to consider a personalized management of acid–base balance disorders based on a comprehensive evaluation of the clinical status of the patient. While the KDIGO guidelines provide a useful framework for treatment, given the complexity of the relationship between SBC and mortality, an individualized approach is required for the management of metabolic acidosis in CKD patients, and clinicians must carefully balance the risks and benefits of acid–base interventions [26].

Further research is needed to fully understand the potential mechanisms underlying this complex relationship in CKD patients. Additionally, future research should explore the potential benefits of interventions aimed at correcting metabolic acidosis in CKD patients, as well as the potential risks associated with overcorrection of SBC levels.

## Supplementary Material

sfae254_Supplemental_File

## Data Availability

The data underlying this article are sensitive health data and cannot be shared publicly due to privacy reasons. The data will be shared on reasonable request to the corresponding author.
