# Supplementary material for: Longitudinal serum bicarbonate and mortality risk in older patients with advanced chronic kidney disease: analyses from the EQUAL cohort
Source: Clin Kidney J. 2024 Aug 22;17(11):sfae254. doi: 10.1093/ckj/sfae254 (PMC11635373; doi:10.1093/ckj/sfae254)

**Supplementary Material**

**Supplementary Tables**

**Supplementary Tables** 1

**Table S1. Cause-specific mortality by study population…** 1

**Table S2. Relationship between the cumulative time-averaged bicarbonate and all-cause mortality in the transition-CKD population…** 2

**Table S3. Relationship between the slope of bicarbonate and all-cause mortality in the transition-CKD population…** 3

**Table S4. Relationship between the cumulative exposure to bicarbonate and all-cause mortality in the transition-CKD population…** 4

**Supplementary Figures** 5

**Figure S1. Continuous relationship between SBC and all-cause mortality in all study populations…** 5

**Figure S2. Estimates and pairwise comparison of the trend in the log hazard of death for selected SBC cut-off points…** 6

**Figure S3. Distribution of SBC levels before and after KRT initiation in the transition-CKD population…** 7

**Figure S4. Continuous relationship between cumulative time-averaged, AUC and slope of SBC and all-cause mortality in the transition CKD population …** 8

**Figure S5 A. Continuous relationship between SBC and all-cause mortality in subgroups…** 9

**Figure S5 B. Continuous relationship between SBC and all-cause mortality in subgroups…** 10

**Table S1. Cause-specific mortality by study population**

| | Whole population N=529 | Pre-KRT population N=345 | KRT population N=176 | Transition CKD population N=177 |
|---|---|---|---|---|
| Cardiac arrest | 54 (15.9) | 29 (13.9) | 20 (18.0) | 25 (20.0) |
| Cerebrovascular accident | 13 ( 3.8) | 7 ( 3.3) | 5 ( 4.5) | 6 ( 4.8) |
| Cachexia | 12 ( 3.5) | 7 ( 3.3) | 5 ( 4.5) | 4 ( 3.2) |
| Heart failure | 51 (15.0) | 37 (17.7) | 6 ( 5.4) | 13 (10.4) |
| Infection | 56 (16.5) | 29 (13.9) | 25 (22.5) | 26 (20.8) |
| Malignancies | 53 (15.6) | 37 (17.7) | 14 (12.6) | 15 (12.0) |
| Myocardial infarction | 29 ( 8.5) | 18 ( 8.6) | 12 (10.8) | 10 ( 8.0) |
| Others | 49 (14.4) | 36 (17.2) | 10 ( 9.0) | 12 ( 9.6) |
| Suicide | 7 ( 2.1) | 3 ( 1.4) | 4 ( 3.6) | 4 ( 3.2) |
| Withdrawal | 16 ( 4.7) | 6 ( 2.9) | 10 ( 9.0) | 10 ( 8.0) |

Missing data 34.9%

**Table S2. Relationship between the cumulative time-averaged bicarbonate and all-cause mortality in the transition-CKD population**

| Q time-averaged SBC mmol/L | Events, N (%) | Person time, years | Events for 1,000 person-years | Unadjusted HR (95% CI) | Sex, age HR (95% CI)[b] | BMI, albumin, SGA HR (95% CI)[b] | eGFR, $K^+$ HR (95% CI)[b] | Comorbidities, primary renal disease HR (95% CI)[b] |
|---|---|---|---|---|---|---|---|---|
| **1st** <20.7 | 52 (39.1) | 272.0 | 191 | 1.01 (0.69, 1.49) p=0.951 | 1.08 (0.73, 1.59) p=0.716 | 1.02 (0.69, 1.51) p=0.932 | 1.06 (0.71, 1.58) p=0.762 | 1.03 (0.69, 1.56) p=0.878 |
| **2nd** 20.7-22.0 | 50 (42.0) | 249.6 | 200 | 1.14 (0.77, 1.67) p=0.514 | 1.12 (0.76, 1.64) p=0.581 | 1.1 (0.75, 1.62) p=0.633 | 1.13 (0.76, 1.66) p=0.554 | 1.17 (0.79, 1.75) p=0.432 |
| **3rd** 22.1-23.7 | 51 (41.5) | 288.6 | 177 | 1.00 (Reference) | 1.00 (Reference) | 1.00 (Reference) | 1.00 (Reference) | 1.00 (Reference) |
| **4th** >23.6 | 60 (47.2) | 256.9 | 234 | 1.33 (0.91, 1.92) p=0.138 | 1.33 (0.92, 1.94) p=0.130 | 1.3 (0.89, 1.89) p=0.172 | 1.3 (0.89, 1.89) p=0.176 | 1.18 (0.79, 1.75) p=0.420 |

Cox regression model; overall study population N= 502; a, quartiles of average-serum bicarbonate; b, HR and 95% CI adjusted sequentially for confounders (sex, age, BMI, SGA, eGFR, $K^+$, comorbidities, primary renal disease).

**Table S3. Relationship between the slope of bicarbonate and all-cause mortality in the transition-CKD population**

| Q<br>Slope of SBC<br>mmol/L per year | Events, N<br>(%) | Person<br>time, years | Events for<br>1,000<br>person-<br>years | Unadjusted<br>HR (95% CI) | Sex, age<br>HR (95% CI)[b] | BMI, albumin,<br>SGA<br>HR (95% CI)[b] | eGFR, $K^+$<br>HR (95% CI)[b] | Comorbidities,<br>primary renal<br>disease<br>HR (95% CI)[b] |
|---|---|---|---|---|---|---|---|---|
| **1st**<br>< -2.4 | 27 (37.0) | 202.2 | 134 | 0.73 (0.42, 1.26)<br>p=0.256 | 0.73 (0.42, 1.26)<br>p=0.256 | 0.71 (0.41, 1.24)<br>p=0.229 | 0.73 (0.42, 1.27)<br>p=0.266 | 0.59 (0.33, 1.06)<br>p=0.079 |
| **2nd**<br>-2.4- -0.1 | 25 (34.7) | 196.8 | 127 | 0.74 (0.43, 1.29)<br>p=0.292 | 0.76 (0.44, 1.31)<br>p=0.322 | 0.76 (0.44, 1.31)<br>p=0.320 | 0.77 (0.44, 1.34)<br>p=0.357 | 0.68 (0.38, 1.22)<br>p=0.193 |
| **3rd**<br>-0.2 - 1.6 | 26 (35.6) | 167.2 | 156 | 1.00 (Reference) | 1.00 (Reference) | 1.00 (Reference) | 1.00 (Reference) | 1.00 (Reference) |
| **4th**<br>>1.6 | 32 (42.1) | 169.1 | 189 | 1.17 (0.7, 1.97)<br>p=0.546 | 1.15 (0.68, 1.93)<br>p=0.598 | 1.14 (0.68, 1.93)<br>p=0.616 | 1.2 (0.69, 2.07)<br>p=0.518 | 1.23 (0.7, 2.18)<br>p=0.470 |

Cox regression model; overall study population N= 294; a, quartiles of serum bicarbonate slope; b, HR and 95% CI adjusted sequentially for confounders (sex, age, BMI, SGA, comorbidities, primary renal disease).

**Table S4. Relationship between the cumulative exposure to bicarbonate and all-cause mortality in the transition-CKD population**

| Q<br>AUC SBC<br>mmol/L per year | Events, N (%) | Person time, years | Events for 1,000 person-years | Unadjusted HR (95% CI) | Sex, age HR (95% CI)[b] | BMI, albumin, SGA HR (95% CI)[b] | eGFR, $K^+$ HR (95% CI)[b] | Comorbidities, primary renal disease HR (95% CI)[b] |
|---|---|---|---|---|---|---|---|---|
| **1st**<br><5.5 | 22 (29.3) | 162.2 | 136 | 2.07 (1.22, 3.54)<br>p=0.007 | 2.05 (1.2, 3.49)<br>p=0.009 | 2.07 (1.21, 3.53)<br>p=0.008 | 2.43 (1.38, 4.26)<br>p=0.002 | 2.44 (1.32, 4.48)<br>p=0.004 |
| **2nd**<br>5.5-6.7 | 35 (47.9) | 174.0 | 201 | 1.35 (0.76, 2.38)<br>p=0.306 | 1.39 (0.78, 2.46)<br>p=0.261 | 1.39 (0.79, 2.47)<br>p=0.256 | 1.43 (0.81, 2.54)<br>p=0.222 | 1.27 (0.69, 2.33)<br>p=0.436 |
| **3rd**<br>6.8-8.5 | 26 (38.2) | 201.6 | 129 | 1.00 (Reference) | 1.00 (Reference) | 1.00 (Reference) | 1.00 (Reference) | 1.00 (Reference) |
| **4th**<br>>8.5 | 27 (35.1) | 195.6 | 138 | 1.38 (0.79, 2.44)<br>p=0.260 | 1.42 (0.8, 2.5)<br>p=0.227 | 1.39 (0.78, 2.46)<br>p=0.259 | 1.31 (0.73, 2.33)<br>p=0.360 | 1.15 (0.63, 2.1)<br>p=0.650 |

Cox regression model; overall study population N= 294; a, quartiles of average-serum bicarbonate; b, HR and 95% CI adjusted sequentially for confounders (sex, age, BMI, SGA, eGFR, $K^+$, comorbidities, primary renal disease).

**Figure S1. Continuous relationship between SBC and all-cause mortality in all study populations.** Multivariable adjusted model, adjusted to age, sex, BMI, SGA, albumin, $K^+$, eGFR, primary renal disease, comorbidities, medications. Whole population p-value nonlinear=0.069; Pre-KRT population, p-value nonlinear=0.029; KRT population p-value nonlinear=0.087; Transition-CKD population p-value nonlinear=0.090.

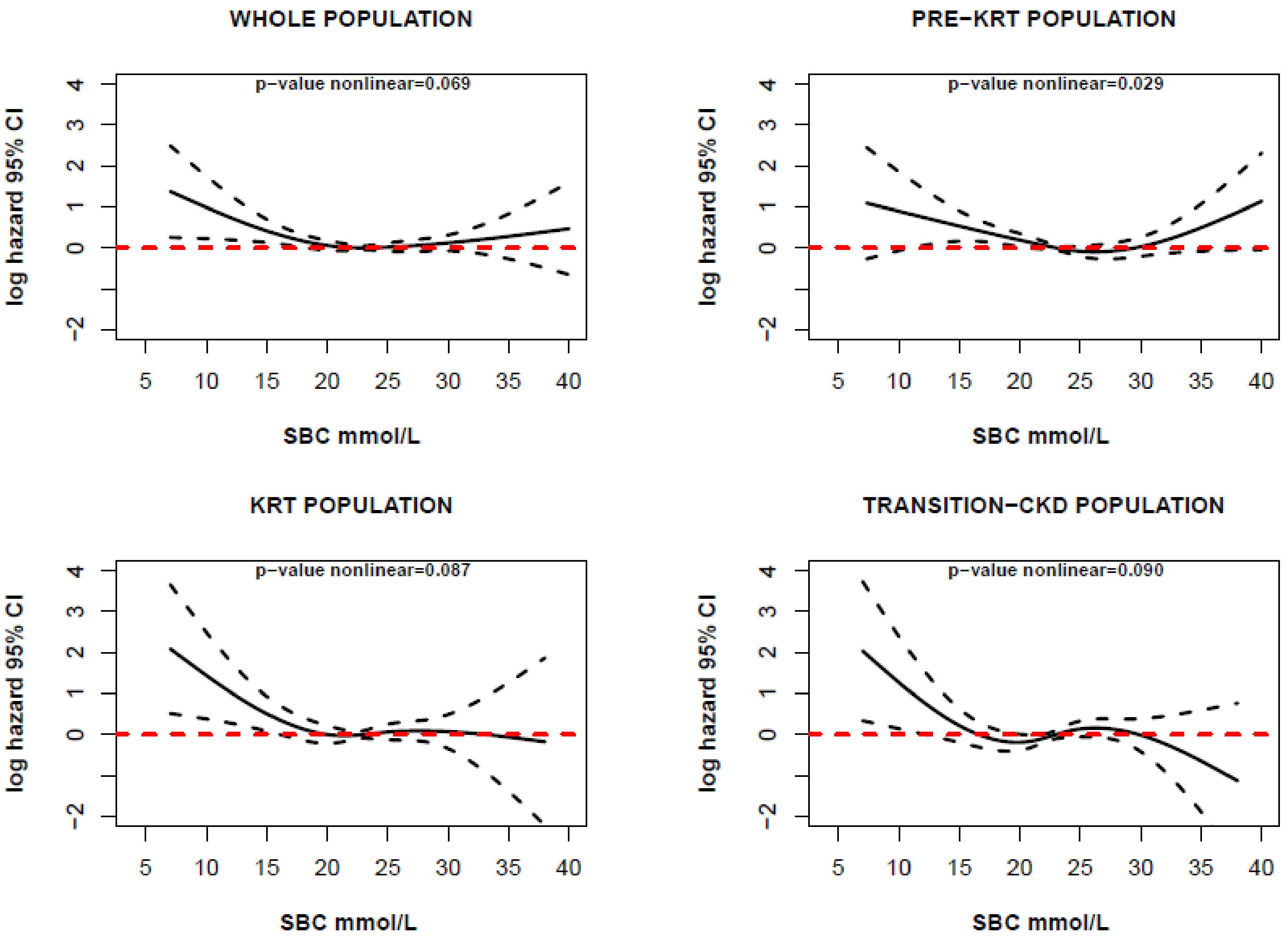

**Figure S2. Estimates and pairwise comparison of the trend in the log hazard of death for selected SBC values.** Trends indicates the change in the log hazard of mortality associated with each SBC cut-off points; adjustments for multiple comparisons were made using the Tukey method

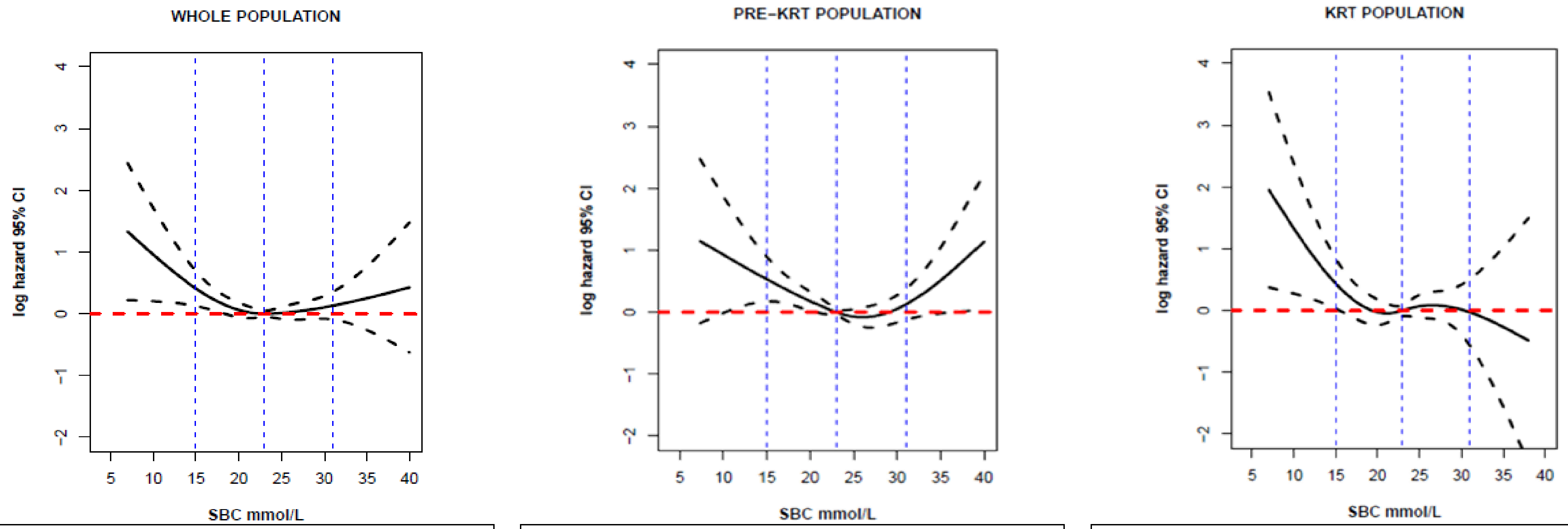


| Estimates of the trend in log hazard of death for selected SBC values in the whole population | | |
|---|---|---|
| **SBC value** | **Slope in log hazard (95% CI)** | **p-value** |
| 15 mmol/L | -0.34 (-0.58, -0.10) | 0.005 |
| 23 mmol/L | 0.01 (-0.06, 0.08) | 0.802 |
| 31 mmol/L | 0.02 (-0.26, 0.30) | 0.890 |

| Estimates of the trend in log hazard of death for selected SBC values in the pre-KRT population | | |
|---|---|---|
| **SBC value** | **Slope in log hazard (95% CI)** | **p-value** |
| 15 mmol/L | -0.23 (-0.55, 0.09) | 0.167 |
| 23 mmol/L | -0.05 (-0.13, 0.04) | 0.302 |
| 31 mmol/L | 0.24 (-0.08, 0.56) | 0.140 |

| Estimates of the trend in log hazard of death for selected SBC values in the KRT population | | |
|---|---|---|
| **SBC value** | **Slope in log hazard (95% CI)** | **p-value** |
| 15 mmol/L | -0.53 (-0.88, -0.18) | 0.003 |
| 23 mmol/L | 0.04 (-0.08, 0.16) | 0.527 |
| 31 mmol/L | -0.32 (-0.87, 0.23) | 0.254 |

| Pairwise comparison of the trends in log hazard of death for selected SBC values in the whole population | | |
|---|---|---|
| **Contrast between SBC values** | **Difference in slope in log hazard (95% CI)** | **p-value** |
| 15 – 23 mmol/L | -0.35 (-0.69, -0.01) | 0.042 |
| 15 – 31 mmol/L | -0.36 (-0.68, -0.03) | 0.027 |
| 23 – 31 mmol/L | -0.01 (-0.38, 0.36) | 0.998 |

| Pairwise comparison of the trends in log hazard of death for selected SBC values in the pre-KRT population | | |
|---|---|---|
| **Contrast between SBC values** | **Difference in slope in log hazard (95% CI)** | **p-value** |
| 15 – 23 mmol/L | -0.18 (-0.63, 0.27) | 0.616 |
| 15 – 31 mmol/L | -0.47 (-0.85, -0.08) | 0.012 |
| 23 – 31 mmol/L | -0.29 (-0.72, 0.15) | 0.278 |

| Pairwise comparison of the trends in log hazard of death for selected SBC values in the KRT population | | |
|---|---|---|
| **Contrast between SBC values** | **Difference in slope in log hazard (95% CI)** | **p-value** |
| 15 – 23 mmol/L | -0.57 (-1.09, -0.05) | 0.027 |
| 15 – 31 mmol/L | -0.21 (-0.84, 0.42) | 0.719 |
| 23 – 31 mmol/L | -0.36 (-0.36, 1.08) | 0.466 |

**Figure S3. Distribution of SBC levels before and after KRT initiation in the transition-CKD population**

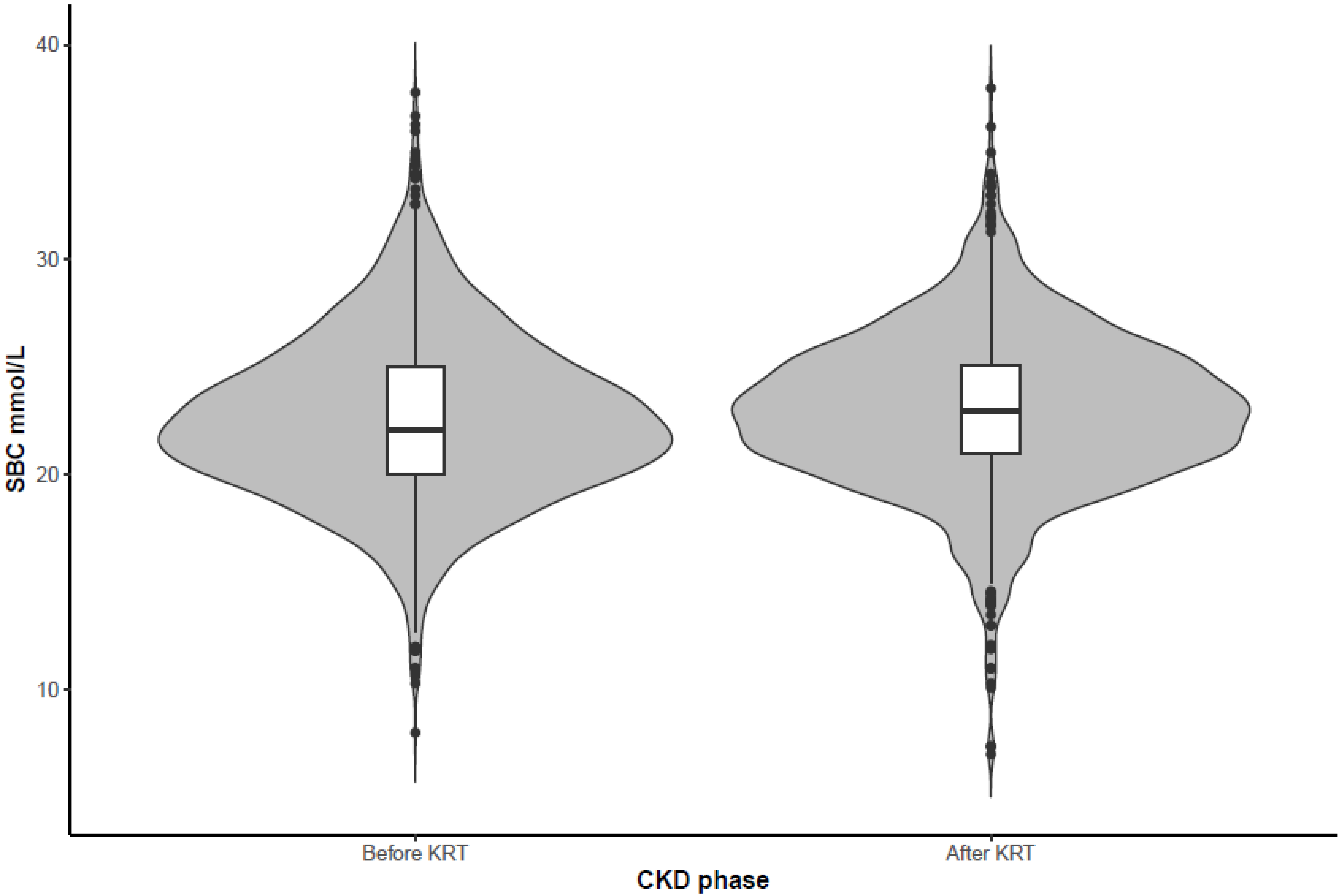

**Figure S4. Continuous relationship between cumulative time-averaged, AUC and slope of SBC and all-cause mortality in the transition CKD population.** Multivariable adjusted model, adjusted to age, sex, BMI, SGA, albumin, $K^+$, eGFR, primary renal disease, comorbidities.

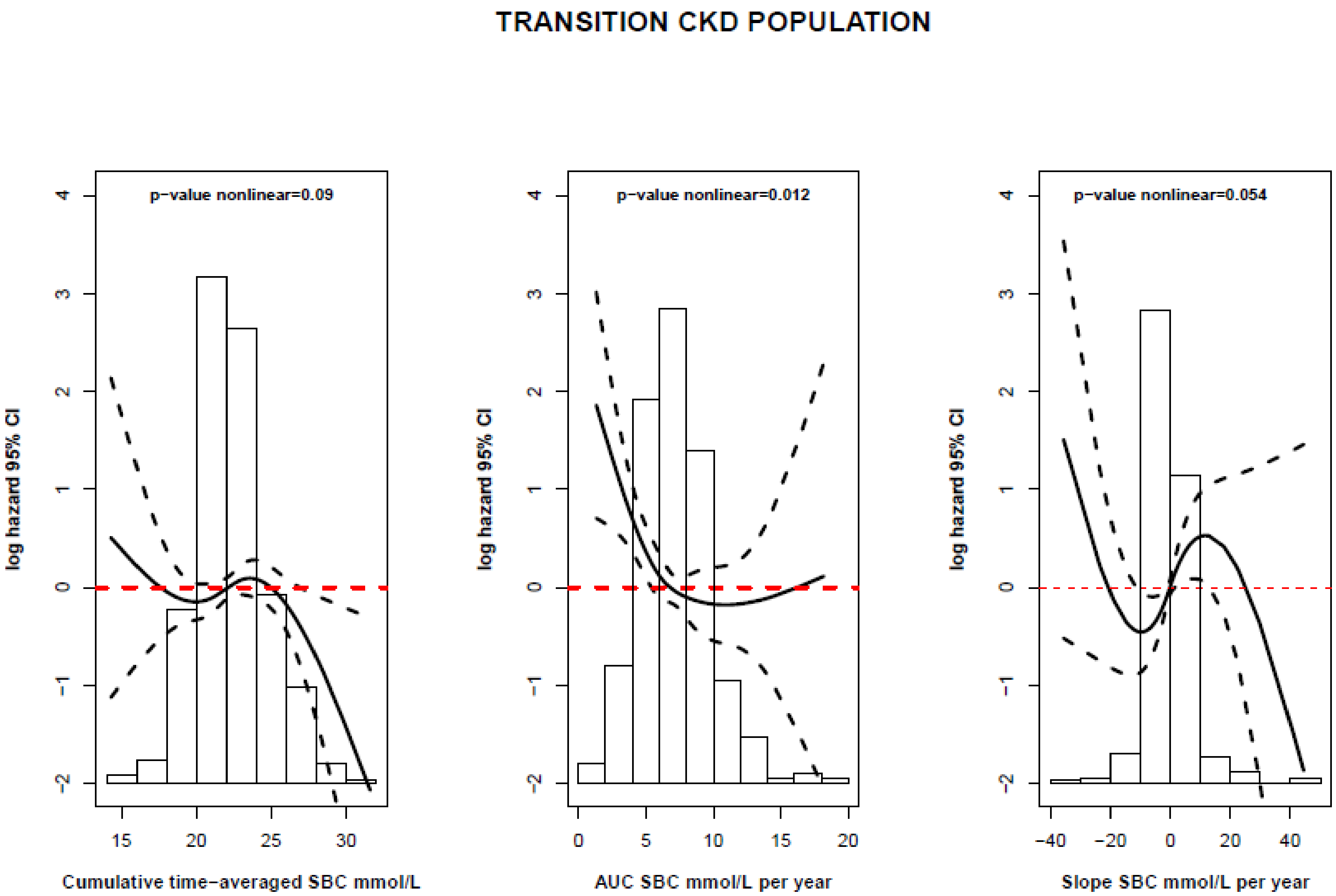

**Figure S5 A. Continuous relationship between SBC and all-cause mortality in subgroups**. Multivariable-adjusted log hazards ratios and 95% CI of all-cause mortality associated with SBC (mmol/L) in select subgroups of patients from the whole population; p-value for interaction SBC x subgroup: age p=0.924, sex p=0.051, BMI p=0.223, albumin p=0.718, SGA p=0.018

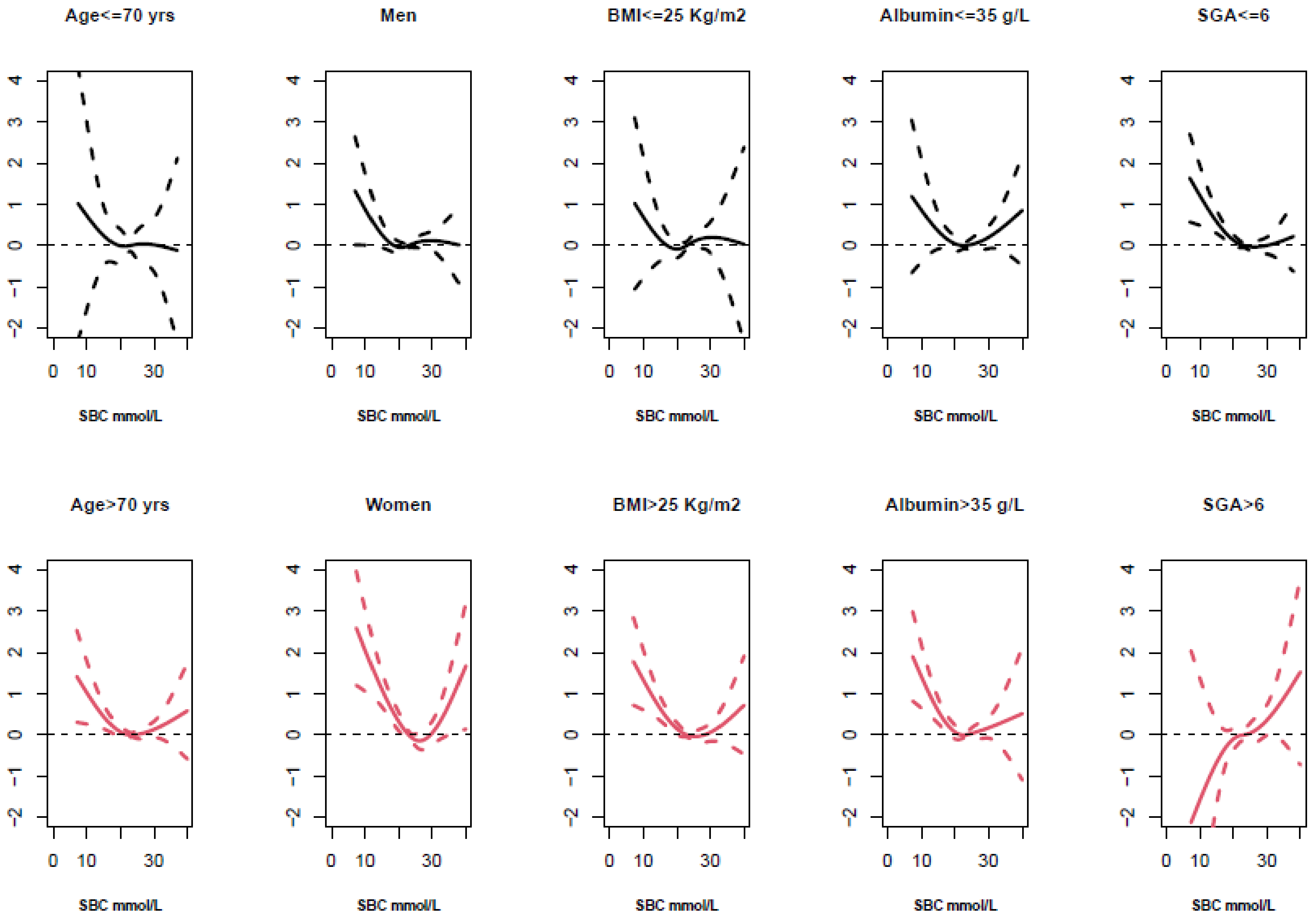

**Figure S5 B. Continuous relationship between SBC and all-cause mortality in subgroups.** Multivariable-adjusted log hazards ratios and 95% CI of all-cause mortality associated with SBC (mmol/L) in select subgroups of patients from the whole population; p-value for interaction SBC x subgroup: cardiovascular diseases p=0.566, diabetes p=0.602, malignancies p=0.163, respiratory diseases p=0.345, KRT initiation p=0.020. Cardiovascular diseases include cerebrovascular disease, coronary artery disease, myocardial infarction and heart failure

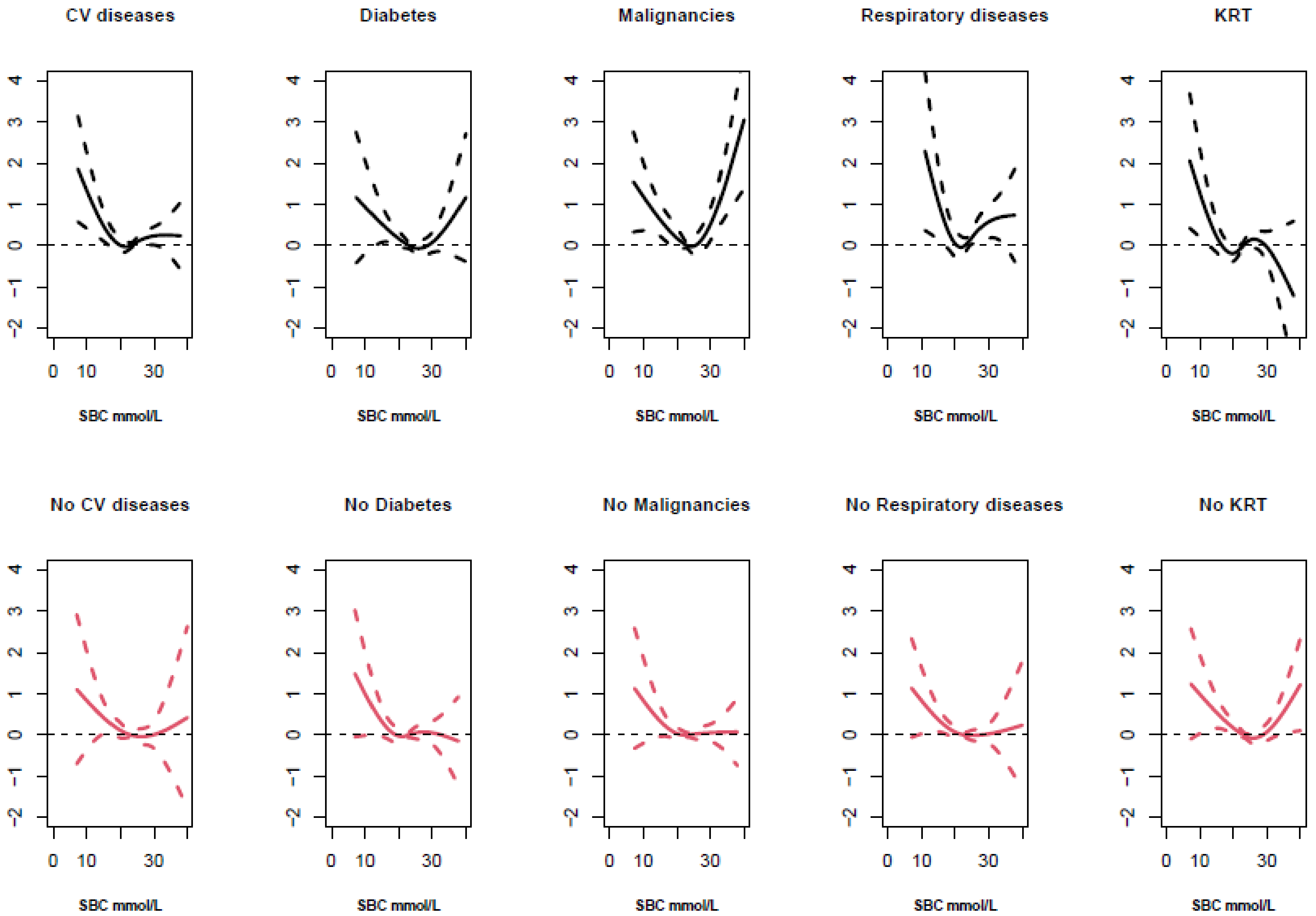

Supplement: sfae254_Supplemental_File [file sfae254_Supplemental_File.pdf]
